# Supplementary material for: Chemical Vapor Deposited Mixed Metal Halide Perovskite Thin Films
Source: Materials (Basel). 2021 Jun 24;14(13):3526. doi: 10.3390/ma14133526 (PMC8269519; doi:10.3390/ma14133526)
Supplement: Supplementary file 1 [file materials-14-03526-s001.zip › materials-1237226-supplementary.pdf]

materials

MDPI

Supplementary Materials: Chemical Vapor Deposited Mixed Metal Halide Perovskite Thin Film

Siphesihle Siphamandla Magubane [1,*], Christopher Joseph Arendse [1,*], Siphelo Ngqoloda [1], Franscious Cummings [1], Christopher Mtshali [2] and Amogelang Sylvester Bolokang [1,3]

1 Department of Physics and Astronomy, University of the Western Cape, Private Bag X17, Bellville 7535, South Africa; sngqoloda@uwc.ac.za (S.N.); fcummings@uwc.ac.za (F.C.); sbolokang@csir.co.za (A.S.B.)
2 iThemba LABS, National Research Foundation, P.O. Box 722, Somerset West 7129, South Africa; mtshali@tlabs.ac.za
3 CSIR Material Science & Manufacturing, Advanced Materials and Engineering, Meiring Naude Road, P.O. Box 395, Pretoria 0001, South Africa
* Correspondence: smagubane@uwc.ac.za (S.S.M.); cjarendse@uwc.ac.za (C.J.A.); Tel.: +27-21-959-3473 (C.J.A.)

Results

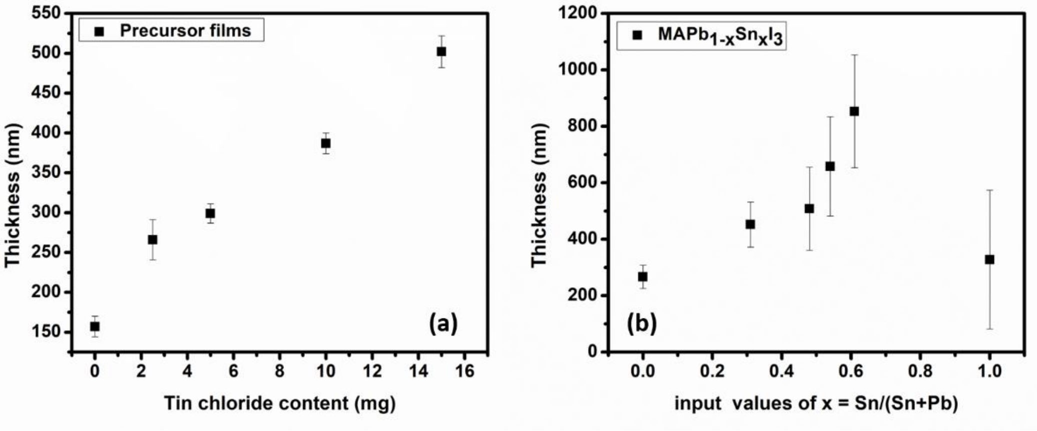

Figure S1. Thickness evolution upon different Sn compositional ratios of (a) precursor films and (b) perovskite films. It is observed from the figure that the thickness of the film increases as the amount of Sn content increases.

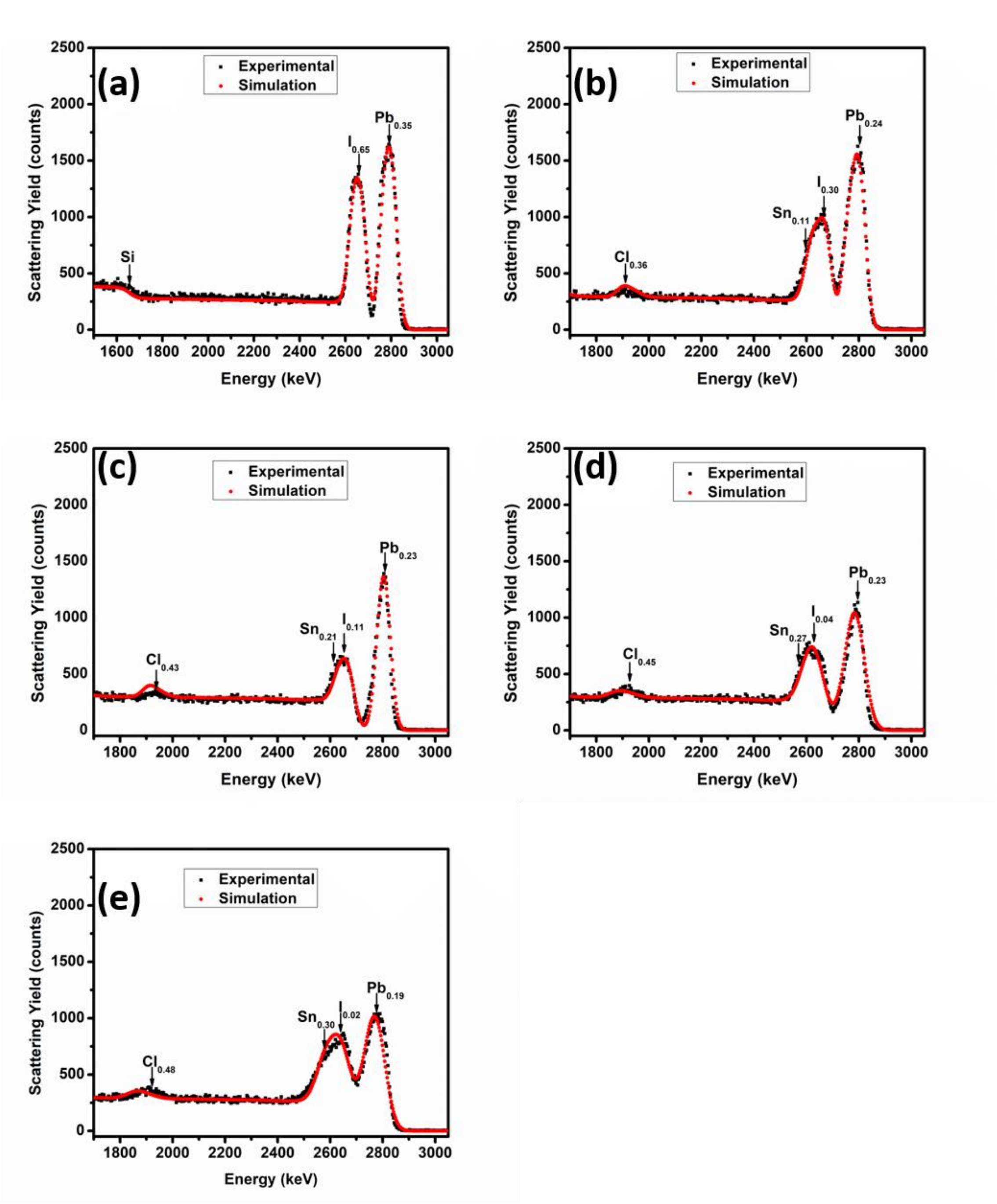

Figure S2. Experimental and simulated RBS spectra of (a) PbI_2 thin film, and (b-e) mixed $SnCl_2$-PbI_2 compound thin films i.e., (2.5: 20) mg, (5: 20) mg, (10: 20) mg and (15: 20) mg, respectively. The simulated spectra in Figure S2 (a) and (b) were obtained using two layers with a total thickness and the average stoichiometry of 3.61×10^{17} atoms/cm^2 and $Pb_{0.35}I_{0.65}$ and 4.22 & $Pb_{0.24}Sn_{0.11}I_{0.30}Cl_{0.36}$, respectively. While the spectra in Figure S2 (c), (d) and (e) were simulated using three layers with a total thickness (×10^{17} atoms/cm^2) and average stoichiometry of, 4.59 & $Pb_{0.23}Sn_{0.21}I_{0.11}Cl_{0.43}$, 4.94 & $Pb_{0.23}Sn_{0.27}I_{0.04}Cl_{0.45}$ and 6.01 & $Pb_{0.19}Sn_{0.30}I_{0.02}Cl_{0.48}$, respectively,.

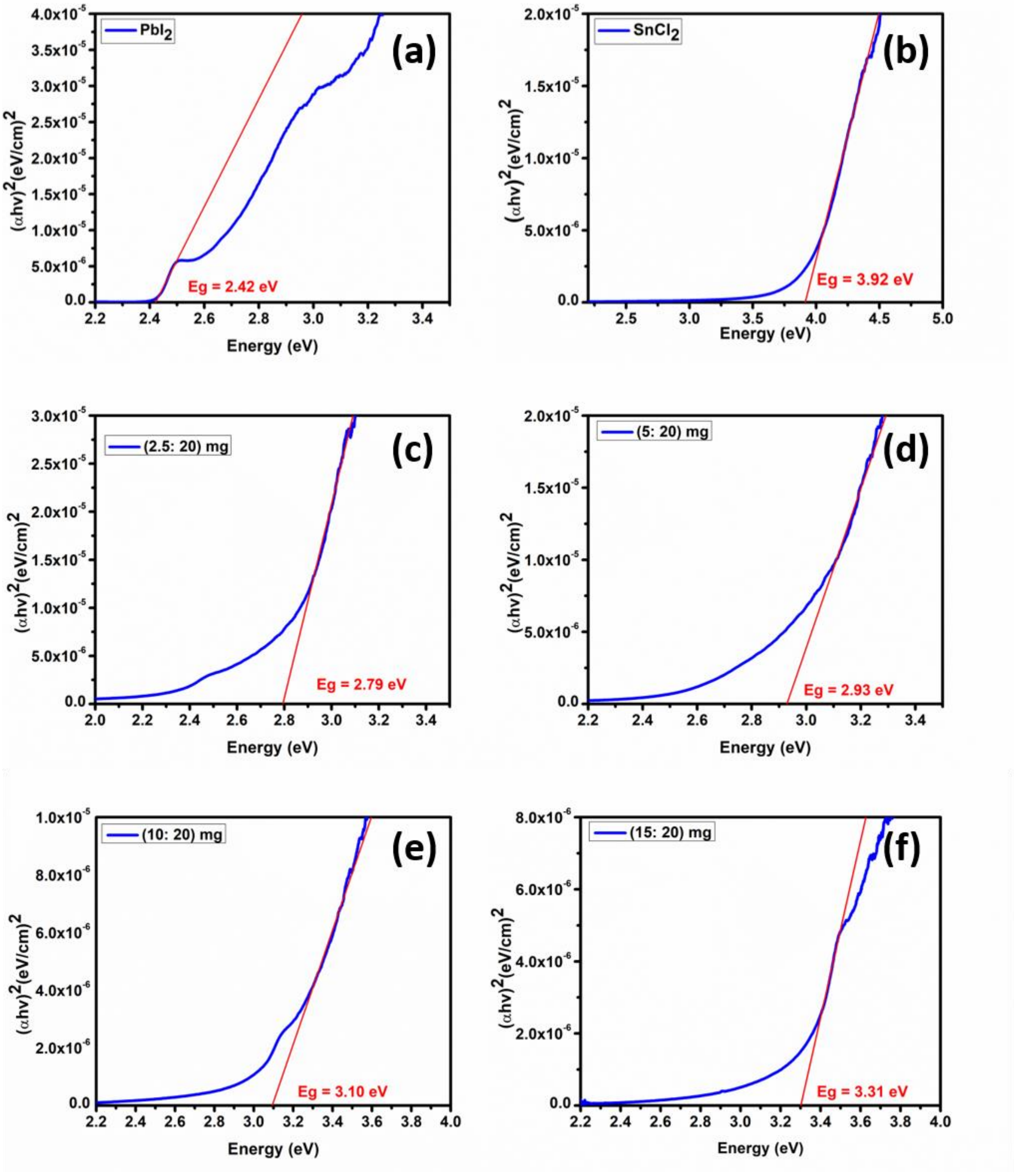

Figure S3. Tauc Plots for the bandgap measurement from the UV-Vis spectra of (a) PbI_2 film, (b) $SnCl_2$ film and (c-f) mixed $SnCl_2$-PbI_2 compound films. The bang gaps of PbI_2 and $SnCl_2$ films were found to be 2.42 eV and 3.92 eV, respectively. While the band gaps of mixed $SnCl_2$-PbI_2 compound films i.e., (2.5: 20) mg, (5: 20) mg, (10: 20) mg and (15: 20) mg were found to be 2.79 eV, 2.93 eV, 3.10 eV and 3.31 eV, respectively.

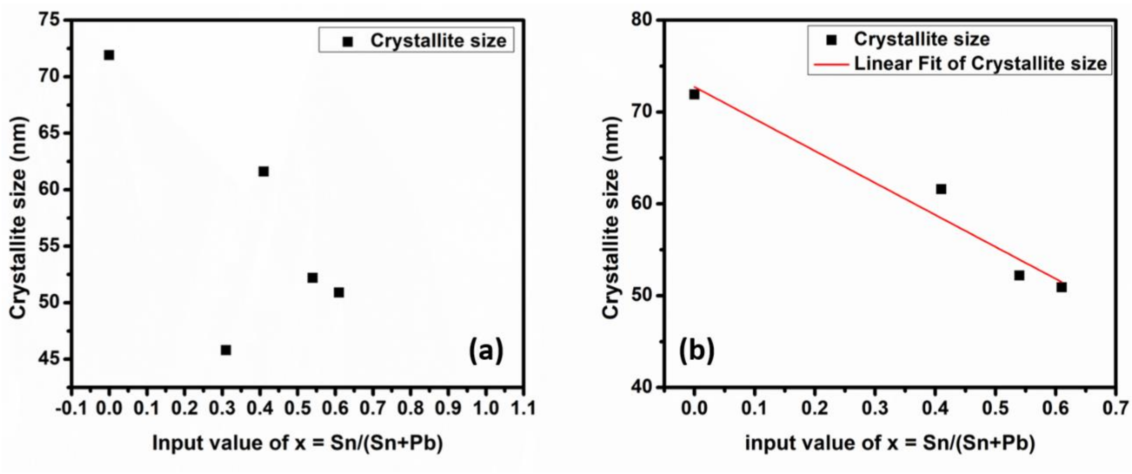

Figure S4. Crystallite size evolution upon increasing the concentration of Sn, (a) includes $MAPbI_3$ and all the mixed metal perovskites. (b) Excludes the 45.8 nm average crystallite size for $MAPb_{0.69}Sn_{0.31}I_3$ perovskite and includes the trendline to guide the eye.

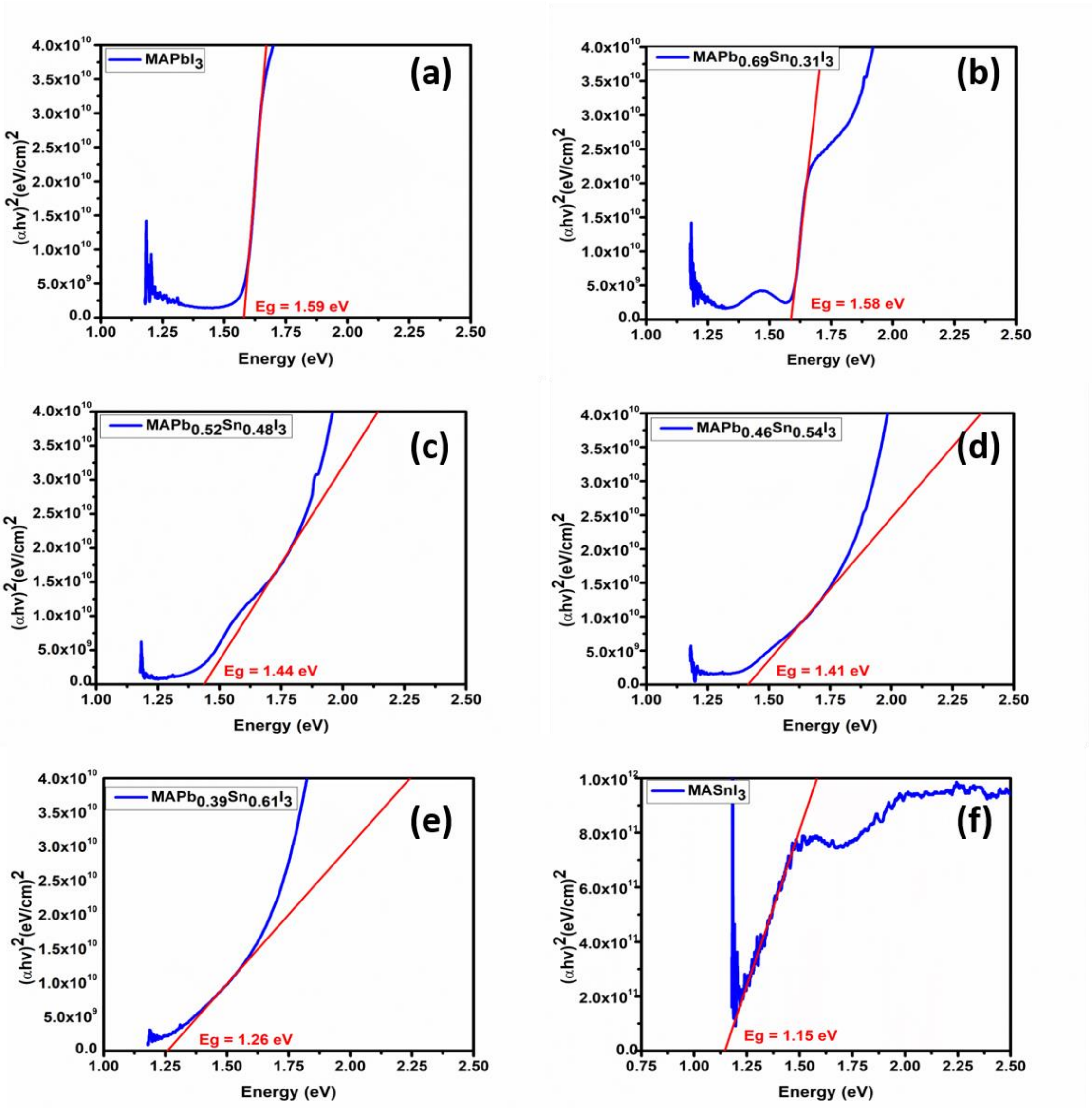

Figure S5: Tauc Plots for the bandgap measurement from the UV-Vis spectra of (a) $MAPbI_3$ film, (b-e) mixed metal perovskite films and (f) $MASnI_3$ film. The bang gaps of $MAPbI_3$ and $MASnI_3$ films were found to be 1.59 eV and 1.15

eV, respectively. While the band gaps of mixed metal perovskite films i.e., $MAPb_{0.69}Sn_{0.31}I_3$, $MAPb_{0.52}Sn_{0.48}I_3$, $MAPb_{0.46}Sn_{0.54}I_3$ and $MAPb_{0.39}Sn_{0.61}I_3$ were found to be 1.58 eV, 1.44 eV, 1.41 eV and 1.26 eV, respectively.

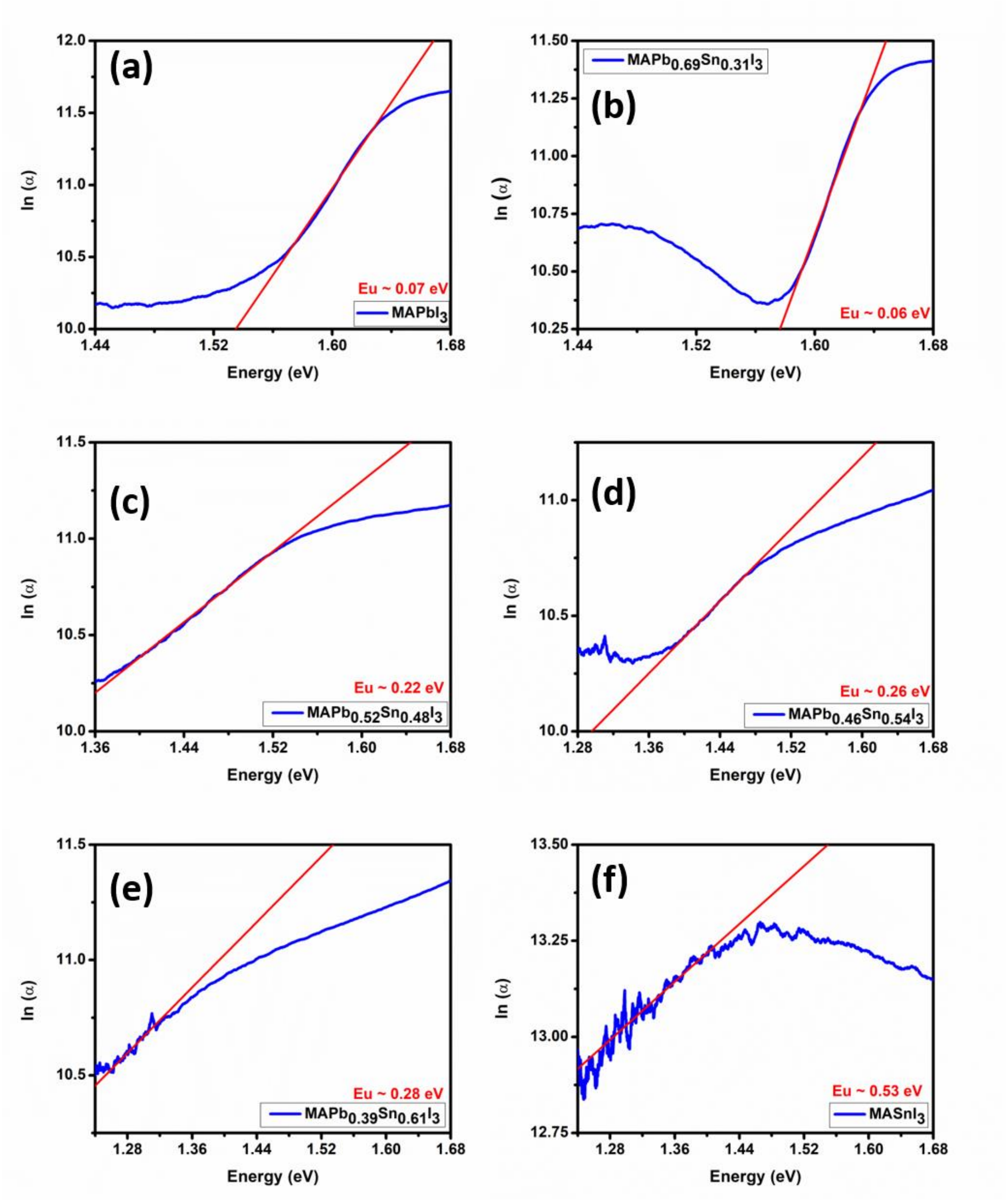

Figure S6. Urbach energy approximation plots of (a) $MAPbI_3$ film, (b-e) mixed metals perovskite films and (f) $MASnI_3$ film. The Urbach energies of $MAPbI_3$ and $MASnI_3$ films were found to be 0.07 eV and 0.53 eV, respectively. While the Urbach energies of mixed metal perovskite films i.e., $MAPb_{0.69}Sn_{0.31}I_3$, $MAPb_{0.52}Sn_{0.48}I_3$, $MAPb_{0.46}Sn_{0.54}I_3$ and $MAPb_{0.39}Sn_{0.61}I_3$ were found to be 0.06 eV, 0.22 eV, 0.26 eV and 0.28 eV, respectively.
